# Foxp3 Represses Retroviral Transcription by Targeting Both NF-κB and CREB Pathways

**DOI:** 10.1371/journal.ppat.0020033

**Published:** 2006-04-28

**Authors:** Christian Grant, Unsong Oh, Kazunori Fugo, Norihiro Takenouchi, Caitlin Griffith, Karen Yao, Timothy E Newhook, Lee Ratner, Steven Jacobson

**Affiliations:** 1 Viral Immunology Section, Neuroimmunology Branch, National Institute of Neurological Disorders and Stroke, National Institutes of Health, Bethesda, Maryland, United States of America; 2 Departments of Medicine, Pathology, and Molecular Microbiology, Washington University School of Medicine, St. Louis, Missouri, United States of America; University of Pennsylvania, United States of America

## Abstract

Forkhead box (Fox)/winged-helix transcription factors regulate multiple aspects of immune responsiveness and Foxp3 is recognized as an essential functional marker of regulatory T cells. Herein we describe downstream signaling pathways targeted by Foxp3 that may negatively impact retroviral pathogenesis. Overexpression of Foxp3 in HEK 293T and purified CD4^+^ T cells resulted in a dose-dependent and time-dependent decrease in basal levels of nuclear factor-κB (NF-κB) activation. Deletion of the carboxyl-terminal forkhead (FKH) domain, critical for nuclear localization and DNA-binding activity, abrogated the ability of Foxp3 to suppress NF-κB activity in HEK 293T cells, but not in Jurkat or primary human CD4^+^ T cells. We further demonstrate that Foxp3 suppressed the transcription of two human retroviral promoters (HIV-1 and human T cell lymphotropic virus type I [HTLV-I]) utilizing NF-κB-dependent and NF-κB-independent mechanisms. Examination of the latter identified the cAMP-responsive element binding protein (CREB) pathway as a target of Foxp3. Finally, comparison of the percent Foxp3^+^CD4^+^CD25^+^ T cells to the HTLV-I proviral load in HTLV-I-infected asymptomatic carriers and patients with HTLV-I-associated myelopathy/tropical spastic paraparesis suggested that high Foxp3 expression is associated with low proviral load and absence of disease. These results suggest an expanded role for Foxp3 in regulating NF-κB- and CREB-dependent cellular and viral gene expression.

## Synopsis

Over the past several years, mounting evidence has shown that immune tolerance in healthy individuals can be maintained by a population of T lymphocytes known as regulatory T cells (Tregs). As a component of this system, a protein known as Foxp3 has been shown to be absolutely required for the development and function of Tregs. While Foxp3 plays an important role in maintaining immune tolerance by blocking T cell proliferation and production of inflammatory proteins known as cytokines, little is known about the molecular mechanisms that are used by Foxp3 to accomplish these events. The present study expands our understanding of how Foxp3 maintains a check on inappropriate immune responses by demonstrating that Foxp3 can block activation of key inducible proteins such as nuclear factor κB (NF-κB) and cAMP-responsive element binding protein (CREB). Since NF-κB and CREB are integrally involved in controlling cell cycle progression, inflammatory cytokine production, and the replication of numerous viruses at the level of transcription, understanding the mechanisms by which Foxp3 functions to regulate cellular and viral gene expression may aid in the discovery of therapeutic approaches designed to rescue the expression and/or function of Foxp3, which have been found to be deficient in several autoimmune diseases and virus-induced disorders.

## Introduction

Immunological tolerance to self-antigens is the result of the deletion of self-reactive T lymphocytes in the thymus (central tolerance) and suppression of the activation of potentially self-reactive T lymphocytes in the periphery (peripheral tolerance) [1]. Suppression of pathogenic T cell responses is mediated by naturally arising CD4^+^CD25^+^ T regulatory cells (Tregs) [2,3]. Deficiencies in Treg development and function have been linked to the severe autoimmune disorder known as immune dysregulation, polyendocrinopathy, enteropathy, X-linked syndrome (IPEX) [4]. In addition, recent studies have provided strong evidence that dysregulation of Treg development and/or function may be a significant factor in the pathogenesis of several autoimmune disorders (e.g., multiple sclerosis [5], myasthenia gravis [6], and type 1 diabetes [7]) and virus-induced immunologic disorders (e.g., human T lymphotropic virus type I [HTLV-I]-associated myelopathy/tropical spastic paraparesis [HAM/TSP], and HIV-induced AIDS [8–10]).

The transcription factor Foxp3 is a 431-amino acid (48-kDa) protein expressed at very high levels in CD4^+^CD25^hi^ T cells and has previously been shown to be absolutely critical for Treg development and function [11–14]. Foxp3 contains a proline-rich amino-terminal domain reported to function as a nuclear factor of activated T cells (NF-AT) and nuclear factor-κB (NF-κB) binding domain, a central region containing a zinc finger and leucine zipper potentially important for protein-protein interactions, and a carboxyl-terminal forkhead (FKH) domain required for nuclear localization and DNA-binding activity [14–16]. Functional inactivation of Foxp3 by genetic mutations affecting the Foxp3 coding region, as demonstrated in IPEX, or repression of Foxp3 expression by the HTLV-I-encoded transactivator protein Tax, as recently reported in patients with HAM/TSP, results in loss of regulatory activity in CD4^+^CD25^hi^ T cells [4,8,17]. Although it is clear that Foxp3 regulates T cell proliferation and cytokine production, very little is known concerning the molecular mechanisms of Foxp3 function.

The first evidence to indicate how Foxp3 promotes the development and function of regulatory T cells came from a report by Ziegler and colleagues [16], which suggested that Foxp3 could inhibit transcriptional activation by physically interacting with forkhead binding sites located immediately adjacent to critical *cis*-acting NF-AT binding sites found in various cytokine promoters (e.g., IL-2 promoter). That study also demonstrated that Foxp3 could repress activation of a synthetic reporter vector containing an SV40 promoter and three tandem copies of a forkhead binding site. These results provided additional evidence suggesting that Foxp3 transcriptional repression was mediated by binding in a sequence-specific manner to promoters containing forkhead binding sites. A recent study by Bettelli and colleagues [15] further demonstrated that Foxp3 could inhibit NF-AT as well as NF-κB activation, although the mechanism of suppression was shown to involve direct protein-protein interactions between NF-AT or NF-κB and Foxp3 rather than binding of Foxp3 to promoter elements adjacent to *cis*-acting NF-AT or NF-κB sites. Collectively, these data suggested that Foxp3 may function as a transcriptional repressor, potentially through the formation of both DNA-protein and protein-protein interactions.

In the present study, we expanded upon these observations by defining additional requirements of Foxp3-mediated repression of NF-κB activation, and investigated whether Foxp3 could target additional signaling pathways by examining transcriptional activation of NF-κB-dependent and NF-κB-independent retroviral pathogens. The characterization of the molecular targets of Foxp3 and the mechanism(s) utilized by Foxp3 to support Treg development and function will aid in our understanding of the role Tregs play in the pathogenesis of human autoimmune disease.

## Results

### Foxp3 Suppresses NF-κB Dependent Transcriptional Activation

**Figure 1:**
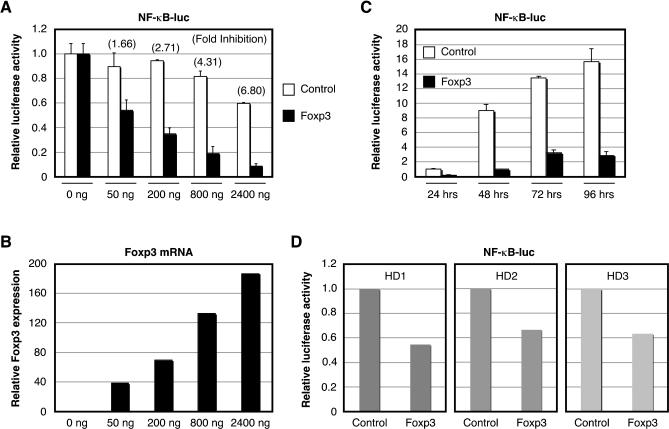
Foxp3 Suppresses NF-κB-Dependent Transcription (A and B) HEK 293T cells (5 × 10^5^) were seeded into six-well plates 1 d prior to transfection with an NF-κB luciferase reporter vector (200 ng) in the presence or absence of indicated concentrations of a Foxp3 expression vector or a control vector and internal reference plasmid (pGL4-TKhRluc2; 50 ng). Cells were harvested 24 h posttransfection and analyzed for luciferase activity (A) and Foxp3 mRNA expression (B). (C) HEK 293T cells (5 × 10^5^) were seeded into six-well plates 1 d prior to transfection with an NF-κB luciferase reporter vector (200 ng) in the presence or absence of a Foxp3 expression vector or a control vector (1,500 ng) and internal reference plasmid (pGL4-TKhRluc2; 50 ng). Cells were harvested at 24, 48, 72, and 96 h posttransfection and analyzed for luciferase activity. (D) CD4^+^ T cells (2 × 10^6^) from three healthy donors were nucleofected with an NF-κB luciferase reporter vector (1,000 ng) and Foxp3 or control expression vectors (2,000 ng) and internal reference plasmid (1,000 ng). Cells were harvested 24 h posttransfection and analyzed for luciferase activity. Relative luciferase activity shown in A, C, and D was normalized to the internal reference control.

To ascertain the molecular mechanisms by which Foxp3 functions to promote the regulatory function of CD4^+^CD25^hi^ T cells, we first confirmed the function of Foxp3 as a repressor of activation of NF-κB, previously implicated as a target of other forkhead/winged-helix family transcription factors (e.g., Foxj1 and Foxo3a) [19,20]. We analyzed the effect of Foxp3 overexpression on NF-κB activation in HEK 293T cells in dose-response and time course analyses. Transfection of HEK 293T cells with an NF-κB luciferase reporter vector in the presence or absence of increasing concentrations of a Foxp3 expression vector or a control vector (enhanced green fluorescent protein [EGFP]) was performed, and cells were harvested after 24 h to assay for luciferase activity and Foxp3 mRNA expression. Results indicated that as the concentration of Foxp3 transfected into cells increases (from 50 to 2,400 ng), the level of NF-κB activation decreases proportionally (Figure 1A). Foxp3 mRNA was also assayed to monitor activity of the Foxp3 expression vector (Figure 1B). Since NF-κB activation was partially affected by transfection of high concentrations of the control vector, we determined the fold inhibition of NF-κB activation by Foxp3 compared to the control vector at each concentration (Figure 1A). Fold inhibition of NF-κB activation was directly proportional to the level of Foxp3 mRNA expression detected by real-time RT-PCR. To determine the level of Foxp3-mediated suppression of NF-κB activation over time, HEK 293T cells were transfected with an NF-κB luciferase reporter vector and an expression vector encoding Foxp3 or EGFP (control vector) and harvested over 4 d. As shown in Figure 1C, NF-κB activation was suppressed by overexpression of Foxp3 at all time points. Extending these results from established, in vitro HEK cell lines to primary human lymphocytes, overexpression of Foxp3 in purified CD4^+^ T cells from three healthy donors also down-regulated the steady-state level of NF-κB activation (Figure 1D). These results recapitulate those from Bettelli and colleagues [15] demonstrating that Foxp3 functions, in part, to block NF-κB-dependent transcription in human cell lines as well as in primary human CD4^+^ T cells.

### The Carboxyl-Terminal FKH Domain Is Not Required for Suppression of NF-κB Activation in T Cells

**Figure 2:**
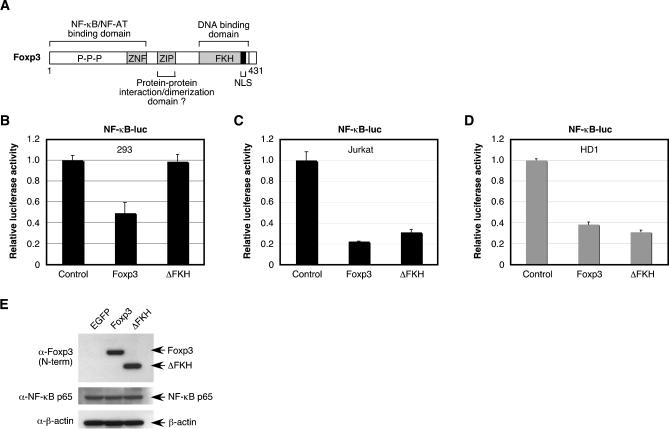
FKH Domain of Foxp3 Is Required to Inhibit NF-κB Activation in HEK 293T Cells but Not CD4^+^ T Cells (A) Schematic representation of human Foxp3, including proline-rich (P-P-P), zinc finger (ZNF), leucine zipper (ZIP), and forkhead (FKH) domains. Mutations within the Foxp3 gene associated with IPEX have been previously described [4,14,43]. The FKH domain contains a nuclear localization signal (NLS), which is required for Foxp3 expression in the nucleus. (B and C) HEK 293T cells (5 × 10^5^) (B) were seeded into six-well plates 1 d prior to transfection or Jurkat T cells (1 × 10^6^) (C) were seeded into six-well plates on the day of transfection with an NF-κB luciferase reporter vector (200 ng) in the presence or absence of a Foxp3, ΔFKH, or control expression vector (1,000 ng) and internal reference plasmid (pGL4-TKhRluc2; 50 ng). Cells were harvested at 24 h posttransfection and analyzed for luciferase activity. Relative luciferase activity was normalized to the internal reference control. (D) CD4^+^ T cells (2 × 10^6^) from healthy donors were nucleofected with an NF-κB reporter plasmid (2,000 ng), together with either a Foxp3, ΔFKH, or control expression vector (2,000 ng), and an internal reference plasmid (1,000 ng). Cells were harvested 24 h posttransfection and analyzed for luciferase activity. Relative luciferase activity was normalized to the internal reference control. Results from one healthy donor are shown. (E) Foxp3, ΔFKH, and NF-κB p65 expression in whole-cell extracts (20 μg) derived from HEK 293T cells transfected with a control expression vector (EGFP), Foxp3, or ΔFKH expression vectors were analyzed by Western blot analysis. β-actin expression was analyzed as a loading control.

To define the requirements of Foxp3 with respect to inhibition of NF-κB-dependent transcription, we utilized a mutant of Foxp3 lacking the FKH domain (Figure 2A) [16], similar to the *scurfy* mutant Foxp3 of mice, and a mutant Foxp3 protein from a patient with IPEX [4,11,14,17]. Unlike full-length Foxp3, which localizes almost exclusively to the nucleus and can bind in a sequence-specific manner to forkhead binding sites, the ΔFKH mutant fails to localize to the nucleus and thus cannot interact with promoter elements or nuclear proteins [16]. Therefore, we utilized the ΔFKH mutant to determine whether nuclear localization (or other function associated with the FKH domain) of Foxp3 was a prerequisite for inhibition of NF-κB activation. Although Foxp3 interaction with NF-κB presumably takes place in the nucleus, it may also be possible for a cytoplasmic Foxp3 protein to bind to NF-κB in the cytoplasm and prevent localization to the nucleus following an activation stimulus. Overexpression of full-length Foxp3, but not of ΔFKH, was able to suppress activation of a cotransfected NF-κB reporter vector in HEK 293T cells (Figure 2B). Both Foxp3 and ΔFKH were expressed at very high levels following transfection as detected by real-time RT-PCR (unpublished data). These data appear to suggest that the carboxyl-terminal FKH domain is critically important for Foxp3 to down-regulate NF-κB-dependent transcription. However, NF-κB activation was blocked to a similar extent by both full-length Foxp3 and ΔFKH in Jurkat T cells (Figure 2C) and primary human CD4^+^ T cells (Figure 2D). Western blot analysis of NF-κB p65 expression demonstrated that Foxp3 and ΔFKH does not block NF-κB activation at the level of p65 protein expression (Figure 2E). These results are very interesting with respect to Foxp3 function, because they suggest that the carboxyl-terminal FKH domain, and possibly nuclear localization, are dispensable for Foxp3 function in T cell populations. Alternative interpretations may include the possibility that the localization of ΔFKH differ between epithelial cells and T cells. In either case, these results suggest a cell type-specific mechanism of action for this Foxp3 mutant.

### Foxp3 Suppresses HIV-1 Gene Expression in Part through Blocking Activation of NF-κB

**Figure 3:**
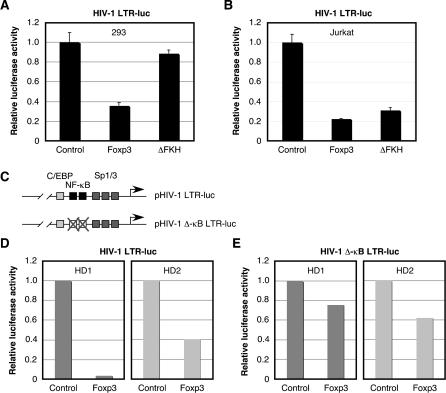
Foxp3 Inhibits Basal Activation of the HIV-1 LTR (A and B) HEK 293T cells (5 × 10^5^) (A) were seeded into six-well plates 1 d prior to transfection or Jurkat T cells (1 × 10^6^) (B) were seeded into six-well plates on the day of transfection with an HIV-1 LTR luciferase reporter vector (200 ng) in the presence or absence of a Foxp3, ΔFKH, or control expression vector (1,000 ng) and internal reference plasmid (pGL4-TKhRluc2; 50 ng). Cells were harvested at 24 h posttransfection and analyzed for luciferase activity. (C) Schematic representation of HIV-1 LTR luciferase reporter constructs pHIV-1 LTR-luc and pHIV-1 Δ-κB LTR-luc. (D and E) CD4^+^ T cells (2 × 10^6^) from two healthy donors were nucleofected with either parental HIV-1 LTR (D) or HIV-1 LTR lacking NF-κB binding sites (at −102 to −81) (E) (1,000 ng), Foxp3 or control expression vectors (2,000 ng), and an internal reference plasmid (1,000 ng). Cells were harvested 24 h posttransfection and analyzed for luciferase activity. Relative luciferase activity shown in A, B, D, and E was normalized to the internal reference control.

If Foxp3 functions as a repressor of NF-κB-dependent gene expression, then we hypothesized that Foxp3 overexpression could selectively down-regulate transcription from promoters previously shown to be responsive to NF-κB. To address this question, we examined the transcriptional activation of the HIV-1 LTR, which contains two tandem *cis*-acting NF-κB binding sites located between positions −102 and −81 with respect to the transcription initiation site [21]. NF-κB plays a crucial role in regulating gene expression directed from the HIV-1 LTR in CD4^+^ T cells [21]. Overexpression of full-length Foxp3, but not ΔFKH, in HEK 293T cells was able to inhibit basal activation of the HIV-1 LTR (Figure 3A), similar to what was previously demonstrated with the synthetic NF-κB reporter vector (Figure 2B). Furthermore, HIV-1 LTR activation was suppressed by full-length Foxp3 and ΔFKH in Jurkat T cells (Figure 3B). To demonstrate that Foxp3-mediated HIV-1 LTR repression was associated with interactions with NF-κB bound to the HIV-1 LTR, we compared basal activation of the HIV-1 LTR or an identical HIV-1 LTR lacking the NF-κB sites located between −102 and −81 (HIV-1 Δ-κB LTR) (Figure 3C). This mutant HIV-1 LTR construct exhibited reduced levels of transcription compared to the parental HIV-1 LTR in purified healthy donor CD4^+^ T cells (unpublished data). However, directly comparing the effect of Foxp3 overexpression on the activation of these two viral promoters demonstrated that Foxp3 was more capable of suppressing transcriptional activation of the HIV-1 LTR (Figure 3D) compared to the mutated HIV-1 LTR (Figure 3E). These results suggest that Foxp3 down-regulation of HIV-1 LTR activation was mediated at least in part by *cis*-acting NF-κB binding sites. Residual levels of inhibition of the HIV-1 Δ-κB LTR by Foxp3 may be due to NF-AT binding sites located upstream of the NF-κB sites within the HIV-1 LTR [22,23].

### The Transactivation Functions of HTLV-I Tax Are Suppressed by Foxp3

**Figure 4:**
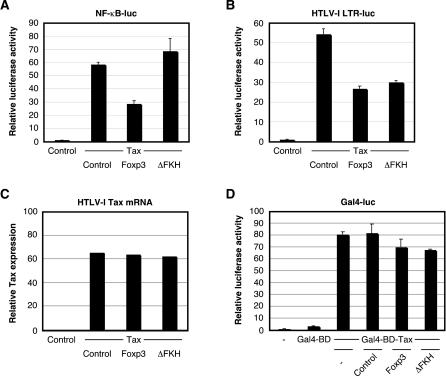
Transactivation Functions of HTLV-I Tax Are Blocked by Overexpression of Foxp3 (A and B) HEK 293T cells (5 × 10^5^) were seeded into six-well plates 1 d prior to transfection with NF-κB (A) or HTLV-I LTR (B) luciferase reporter vectors (200 ng) in the presence or absence of an HTLV-I Tax, Foxp3, ΔFKH, or control expression vector (500 ng) and internal reference plasmid (pGL4-TKhRluc2; 50 ng). Cells were harvested at 24 h posttransfection and analyzed for luciferase activity. Relative luciferase activity was normalized to the internal reference control. (C) Expression of HTLV-I Tax in (A) and (B) was measured by quantitative RT-PCR (TaqMan) to demonstrate that Foxp3 did not suppress Tax transactivation of NF-κB and the HTLV-I LTR by down-regulating the expression of Tax. (D) HEK 293T cells (5 × 10^5^) were seeded into six-well plates 1 d prior to transfection with a Gal4 luciferase reporter vector (200 ng) in the presence or absence of a Gal4-BD or Gal4-BD-Tax expression vector (500 ng), Foxp3, ΔFKH, or control expression vector (500 ng) and internal reference plasmid (pGL4-TKhRluc2; 50 ng). Cells were harvested at 24 h posttransfection and analyzed for luciferase activity. Relative luciferase activity was normalized to the internal reference control.

Previous studies by Bettelli and colleagues have demonstrated that Foxp3 can repress both the basal levels of NF-κB activation as well as tumor necrosis factor-α-stimulated NF-κB activation [15]. Our next step was to determine whether Foxp3 could also suppress the activation of NF-κB caused by a strong viral transactivator protein. HTLV-I encodes a multifunctional transactivator protein, Tax, capable of activating both the NF-κB and CREB pathways [24–29]. Since the HTLV-I Tax protein can function at multiple levels in both the cytoplasm and the nucleus to stimulate activation of NF-κB [28,29], we hypothesized that overexpression of Foxp3 may interfere with this process. However, since Tax-dependent HTLV-I gene expression is independent of NF-κB [18], we also hypothesized that Foxp3 would not affect Tax-dependent activation of the HTLV-I LTR. To test these hypotheses, we overexpressed HTLV-I Tax, full-length Foxp3, and/or ΔFKH in HEK 293T cells cotransfected with an HTLV-I LTR or NF-κB reporter vector. As shown in Figure 4A, HTLV-I Tax strongly up-regulated NF-κB-dependent transcriptional activation (~60-fold). Interestingly, overexpression of Foxp3, but not ΔFKH, suppressed Tax-mediated activation of NF-κB-dependent transcription. These observations further suggest that the carboxyl-terminal FKH domain is required for inhibiting activation of NF-κB in the presence of Tax in HEK 293T cells, strikingly similar to the requirements of Foxp3 inhibition of basal NF-κB activation shown in Figure 2B. Transactivation of the HTLV-I LTR was stimulated about 55-fold by overexpression of Tax (Figure 4B), while transfection of Foxp3 suppressed Tax-dependent HTLV-I LTR activation, although HTLV-I LTR activation in the presence or absence of Tax is independent of NF-κB or NF-AT (another transcriptional activator known to interact with Foxp3). Furthermore, overexpression of ΔFKH also led to suppression of HTLV-I transactivation by Tax to a similar extent as full-length Foxp3 (Figure 4B). The suppressive effects shown in Figure 4A and 4B were not the result of Foxp3 down-regulating the expression of the transfected Tax plasmid as determined by real-time RT-PCR (Figure 4C). These results strongly suggest that Foxp3 interacts with transcriptional regulators in addition to NF-κB and NF-AT, and that the carboxyl-terminal FKH domain, and therefore localization to the nucleus, are not required for inhibition of Tax-mediated HTLV-I LTR activation (even in HEK 293T cells).

**Figure 5:**
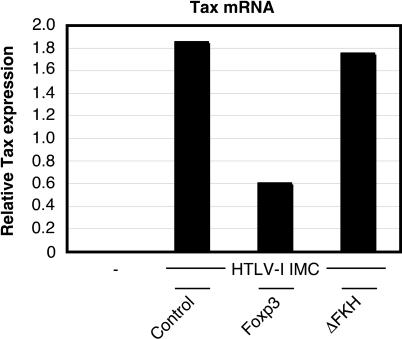
Foxp3 Inhibits the Expression of HTLV-I Tax Directed from an Infectious Molecular Clone HEK 293T cells (5 × 10^5^) were seeded into six-well plates 1 d prior to transfection in the absence or presence of an HTLV-I infectious molecular clone (pACH; 800 ng) and either a control (EGFP), Foxp3, or ΔFKH expression vector (800 ng). Expression of HTLV-I Tax from the molecular clone was measured by quantitative RT-PCR (TaqMan).

To determine whether Foxp3 inhibited the transactivation functions of Tax by directly associating with this viral protein, we generated an expression vector in which HTLV-I Tax was fused in-frame to the carboxyl terminus of the Gal4 DNA-binding domain (Gal4-BD). This Gal4-BD-Tax fusion protein activated transcription of a synthetic promoter containing five Gal4 binding sites, while Gal4-BD was insufficient to stimulate transcription by itself (Figure 4D). Transactivation of the Gal4-resposive promoter by Gal4-BD-Tax remained relatively unaffected by overexpression of either EGFP (control), Foxp3, or ΔFKH, suggesting that Foxp3 does not repress Tax transactivation by directly interfacing with the HTLV-I Tax protein. To confirm that Foxp3 had a direct effect on HTLV-I replication, we transfected HEK 293T cells with a well-characterized HTLV-I infectious molecular clone (termed ACH) [30] in the presence of full-length Foxp3, ΔFKH, or control vector. ACH has been previously shown to direct the expression of viral antigens, produce infectious virus, and transform CD4^+^ T cells both in vitro and in vivo [31,32]. After 24 h, the amount of viral antigen expression, in this case Tax mRNA, was detected by a sensitive real-time RT-PCR assay. As illustrated in Figure 5, the level of Tax mRNA synthesized from ACH was down-regulated in the presence of Foxp3 compared to the level produced in the presence of the control vector. ΔFKH did not have a discernable affect on Tax expression. These data indicate that Foxp3 is capable of repressing the expression of Tax from an infectious HTLV-I molecular clone.

### Increased Foxp3 Protein Expression Is Associated with Low HTLV-I Proviral Load

Since Foxp3 is expressed almost exclusively within CD4^+^CD25^+^ T cells, a major viral reservoir for HTLV-I [33], it was important to determine whether there was an association between Foxp3 and HTLV-I replication in infected patients. We therefore quantitated the Foxp3 protein expression in CD4^+^CD25^+^ T cells by flow cytometry and the HTLV-I proviral load (a surrogate marker of viral replication) by real-time PCR from eight patients with HAM/TSP and eight asymptomatic carriers (ACs). The data from this analysis is summarized in Table 1. As expected, patients with HAM/TSP exhibited significantly higher proviral loads (indicated as HTLV-I proviral DNA copies/100 cells) (34.68 ± 23.19) compared to ACs (4.75 ± 5.47) (*p* = 0.0008). The percentage of Foxp3^+^ cells within the CD4^+^CD25^+^ T cell population was significantly greater in ACs (43.23 ± 12.95) than in HAM/TSP patients (18.59 ± 5.77) (*p* = 0.0033). These data suggest that high levels of Foxp3 protein expression are associated with reduced HTLV-I replication in vivo. They also support our recent reports that high proviral loads, which have been shown to correlate with high Tax mRNA in HTLV-I-infected patients, are associated with reduced Foxp3 expression [8,34].

**Table 1:**
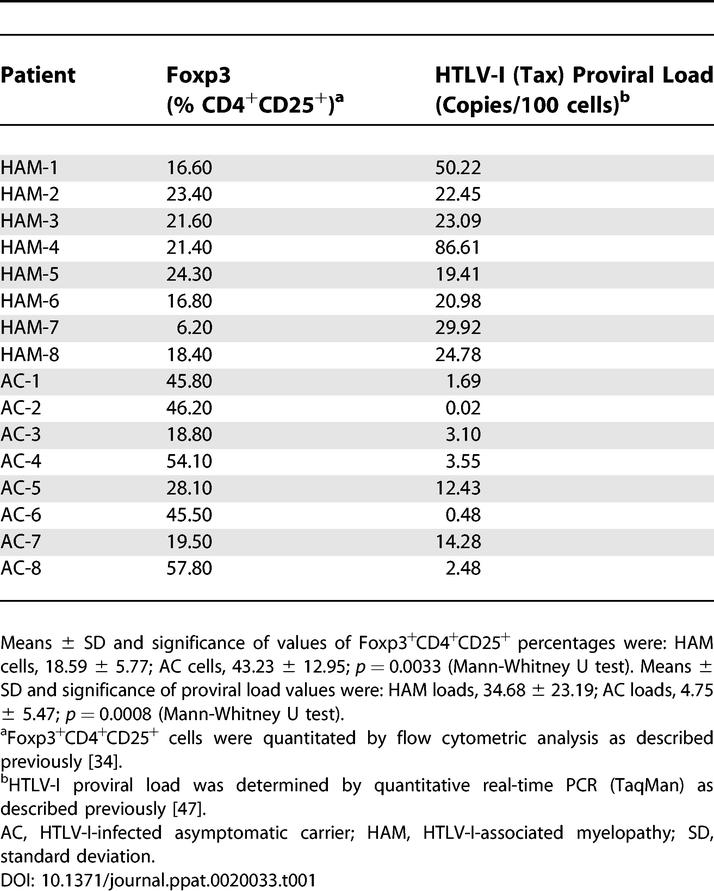
Expression of Foxp3 in CD4^+^CD25^+^ T Cells in HTLV-I-Infected Patients

### CREB Is a Target for Transcriptional Repression by Foxp3

**Figure 6:**
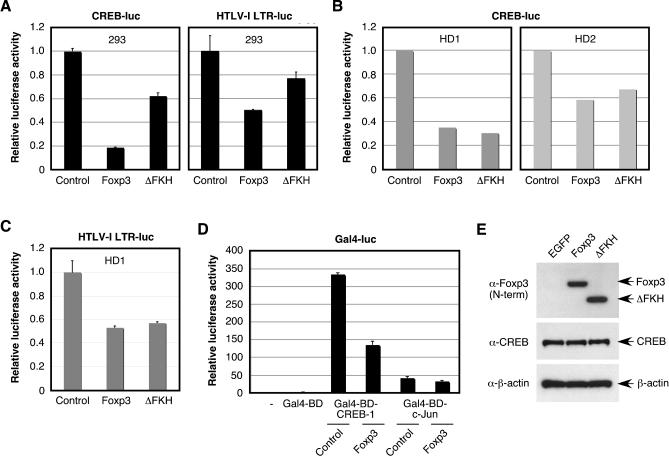
Foxp3 Inhibits CREB-Dependent Transcription (A) HEK 293T cells (5 × 10^5^) were seeded into six-well plates 1 d prior to transfection with a CREB or an HTLV-I LTR luciferase reporter vector (200 ng) in the presence or absence of a Foxp3, ΔFKH, or control expression vector (500 ng) and internal reference plasmid (pGL4-TKhRluc2; 50 ng). (B) Purified CD4^+^ T cells (2 × 10^6^) from two healthy donors were transfected with a CREB luciferase reporter vector (2,000 ng) in the presence or absence of a Foxp3, ΔFKH, or control expression vector (2,000 ng) and internal reference plasmid (pGL4-TKhRluc2; 1,000 ng). (C) Purified CD4^+^ T cells (2 × 10^6^) from healthy donors were transfected with an HTLV-I LTR luciferase reporter vector (2,000 ng) in the presence or absence of a Foxp3, ΔFKH, or control expression vector (2,000 ng) and internal reference plasmid (pGL4-TKhRluc2; 1,000 ng). (D) HEK 293T cells (5 × 10^5^) were seeded into six-well plates 1 d prior to transfection with a Gal4 luciferase reporter vector (200 ng) in the presence or absence of Gal4-BD, Gal4-BD-CREB-1, or Gal4-BD-c-Jun (500 ng) along with Foxp3 or control expression vector (500 ng) and internal reference plasmid (pGL4-TKhRluc2; 50 ng). Cells from A, B, C, and D were harvested at 24 h posttransfection and analyzed for luciferase activity. Relative luciferase activity was normalized to the internal reference control. (E) Foxp3, ΔFKH, and CREB-1 expression in whole-cell extracts (20 μg) derived from HEK 293T cells transfected with a control expression vector (EGFP), Foxp3, or ΔFKH expression vectors were analyzed by Western blot analysis. β-actin expression was analyzed as a loading control.

Although Foxp3 could down-regulate Tax-dependent transactivation of the HTLV-I LTR (Figure 4B) and inhibit Tax expression from an infectious molecular clone (Figure 5), Foxp3 failed to modulate Tax function in the absence of the viral promoter (Figure 4D). These results led us to hypothesize that Foxp3 acts on HTLV-I gene expression by interacting with proteins important for driving HTLV-I LTR activity in vivo. Previous studies have demonstrated that the Tax-responsive elements within the HTLV-I LTR play a crucial role in driving Tax-mediated transactivation of the HTLV-I LTR [18]. The Tax-responsive elements have been shown to resemble CREB binding sites, bind CREB in vitro and in vivo, and facilitate HTLV-I LTR activation both in the presence and in the absence of Tax [35,36]. Ching and colleagues [18] demonstrated that addition of a dominant-negative CREB expression vector resulted in nearly complete inhibition of Tax-mediated activation of the HTLV-I LTR, while blocking NF-κB activation by addition of a dominant-negative IKKβ expression vector had no effect on Tax transactivation of the HTLV-I LTR. Therefore, we hypothesized that Foxp3 may inhibit Tax transactivation of the HTLV-I LTR via disruption of the CREB signaling pathway. To test this possibility, HEK 293T cells were transfected with an HTLV-I LTR or synthetic CREB reporter vector along with a control expression vector (EGFP) or expression vectors encoding Foxp3 or ΔFKH. As shown in Figure 6A, Foxp3 down-regulated basal activation of the HTLV-I LTR and transcription of a synthetic CREB reporter vector, suggesting that Foxp3 down-regulates HTLV-I LTR activation by targeting the CREB pathway. Deletion of the FKH domain of Foxp3 dampened the suppressive effect of Foxp3, but did not completely abrogate suppression, as is seen with NF-κB-responsive promoters in HEK 293T cells. Like NF-κB activation, CREB transcriptional activation was also suppressed by expression of Foxp3, and to a similar extent ΔFKH, in healthy donor CD4^+^ T cells (Figure 6B). Similarly, Foxp3 and ΔFKH also repressed basal HTLV-I LTR activation in primary human CD4^+^ T cells (Figure 6C). To our knowledge, this is the first evidence implicating CREB as a molecular target of Foxp3. As observed with NF-κB activation, ΔFKH was a more potent inhibitor of CREB activation in CD4^+^ T cells than in HEK 293T cells, further indicating that a cell type-specific mechanism of action may govern the function of this Foxp3 mutant.

To determine whether Foxp3 functioned by directly signaling through CREB, we utilized expression vectors encoding CREB-1 or c-Jun (a member of the activator protein 1 family of transcription factors) fused in-frame to the Gal4-BD (Gal4-BD-CREB-1 and Gal4-BD-c-Jun). As shown in Figure 6D, activation of a Gal4-responsive reporter vector by Gal4-BD-CREB-1 was down-regulated by Foxp3 compared to control vector (EGFP), indicating that Foxp3 functions by directly or indirectly interacting with CREB-1. However, Foxp3 failed to markedly affect transcriptional activation of Gal4-BD-c-Jun (c-Jun has also been demonstrated to bind to the HTLV-I LTR) and Gal4-BD-Tax (see Figure 5). Importantly, the mechanism of Foxp3-mediated inhibition of CREB-dependent transcription was not due to a block in CREB-1 protein expression, as determined by Western blot analysis (Figure 6E). Although these results demonstrate that Foxp3 functions as a co-repressor of CREB activation (in addition to NF-κB and NF-AT), we were unable to detect a direct physical interaction between CREB-1 and Foxp3 by coimmunoprecipitation or mammalian two-hybrid analysis (unpublished data). Therefore, our data suggest that Foxp3 may interfere with CREB signaling at an upstream event, such as phosphorylation of CREB or recruitment/function of coactivator proteins CREB-binding protein (CBP)/p300.

### Foxp3 Antagonizes CREB Transcriptional Activation by Disrupting Coactivator Recruitment

**Figure 7:**
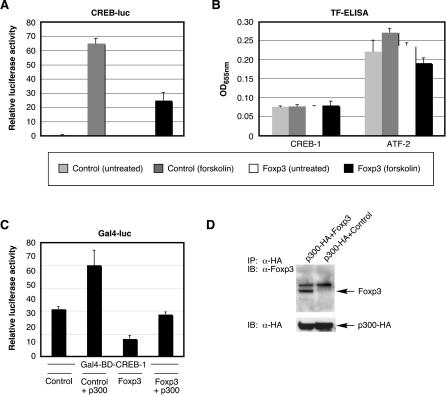
Foxp3 Antagonizes CREB Activation by Blocking Recruitment of Coactivator Protein p300 (A and B) HEK 293T cells (5 × 10^5^) were seeded into six-well plates 1 d prior to transfection with a CREB or an HTLV-I LTR luciferase reporter vector (200 ng) in the presence or absence of a Foxp3, DFKH, or control expression vector (500 ng) and internal reference plasmid (pGL4-TKhRluc2; 50 ng). Forskolin (10 μM) was added to the appropriate reactions 20 h posttransfection. Reactions were assayed 24 h posttransfection for luciferase activity (A) and CREB-1 and ATF-2 DNA-binding activity (B). (C) HEK 293T cells (5 × 10^5^) were seeded into six-well plates 1 d prior to transfection with a Gal4 luciferase reporter vector (200 ng) in the presence or absence of Gal4-BD-CREB-1 (500 ng) along with a control, Foxp3, or p300 expression vector (500 ng) and internal reference plasmid (pGL4-TKhRluc2; 50 ng). Cells were harvested at 24 h posttransfection and analyzed for luciferase activity. Relative luciferase activity in (A and C) was normalized to the internal reference control. (D) Whole-cell extracts from HEK 293T cells transfected with a control (EGFP) or Foxp3 expression vector together with a p300-HA expression vector were immunoprecipitated with anti-HA monoclonal antibody. Proteins were then separated by SDS-PAGE and immunoblotted with anti-Foxp3 (ab10563) to detect immunoprecipitates and with anti-HA for the lysates.

Stimulation of CREB-dependent transcription by reagents that activate adenylate cyclase and increase cAMP levels (e.g., forskolin) increase the transactivation potential of CREB through phosphorylation of serine 133 by protein kinase A, which permits binding and recruitment of coactivators CBP/p300 to the promoter [37,38]. Phosphorylation of serine 133 does not, however, affect the DNA-binding activity of CREB in most cases [39–41]. Addition of forskolin to HEK 293T cells stimulated activation of a CREB reporter vector about 65 fold (Figure 7A). Overexpression of Foxp3 was capable of down-regulating forskolin-induced CREB transcriptional activation. The functional interaction between Foxp3 and CREB did not affect the DNA-binding activity of CREB-1, but did show a modest decrease in activating transcription factor 2 (ATF-2) DNA-binding activity in the presence of forskolin as determined by transcription factor ELISA (Figure 7B).

While Foxp3 has been shown to bind to and repress activation of both NF-κB and NF-AT, exactly how Foxp3 functions to bring about this affect has not been elucidated. To determine how Foxp3 blocks CREB-dependent transcription, we examined whether Foxp3 was capable of (1) disrupting the recruitment of coactivator proteins and/or (2) preventing phosphorylation of CREB at serine 133 (which is a prerequisite for coactivator recruitment). Since both of these events are required for CREB-dependent gene expression, we hypothesized that Foxp3 may affect CREB activation at both steps. To determine whether Foxp3 can disrupt the function/recruitment of the coactivator protein p300, we introduced a Gal4 reporter vector and a Gal4-BD-CREB-1 expression vector into HEK 293T cells in the absence or presence of Foxp3, p300, and/or control expression vectors (Figure 7C). As expected, p300 overexpression stimulated transcription of the Gal4-BD-CREB-1 fusion protein. Foxp3, again, repressed basal levels of Gal4-BD-CREB-1 activation by more than 2-fold, while effectively neutralizing Gal4-BD-CREB-1 activation in the presence of p300. We next analyzed the effect of Foxp3 on phosphorylation of CREB at serine 133 in forskolin-treated HEK 293T cells by Western blot analysis. Overexpression of Foxp3 failed to reduce the detectable levels of CREB phosphorylation using a phosphospecific antibody for CREB-1 (unpublished data). However, when we attempted to determine whether Foxp3 could physically interact with the coactivator protein p300, we found that p300 immunoprecipitated Foxp3 when both proteins were overexpressed in HEK 293T cells (Figure 7D). Collectively, these results suggest that Foxp3 antagonizes CREB-dependent gene expression by directly interacting with coactivator p300 and interfering with its function and/or recruitment to CREB-responsive promoter sequences.

## Discussion

In the present study, we show that Foxp3 functions as a potent repressor of NF-κB- and CREB-dependent transcriptional activation. Furthermore, the carboxyl-terminal FKH domain appears to be dispensable for mediating these effects, at least in T cell populations. This observation may become important in light of recent reports suggesting that Foxp3 expression in thymic epithelial cells was crucial for directing development of T cells in the thymus [42]. Interestingly, the majority of the genetic mutations associated with IPEX, a severe autoimmune disorder caused by functional inactivation of Foxp3, map to the carboxyl-terminal FKH domain or the leucine zipper domain in the central region of the protein. Only one mutation associated with IPEX to date has been mapped to the amino-terminal proline-rich region [43]. It is possible that the FKH domain has a complex tertiary structure that is particularly sensitive to misfolding caused by genetic mutations and that an intact FKH domain is absolutely critical for promoting Foxp3 function in the nucleus, whereas the structure of the amino-terminal proline-rich region may tolerate certain mutations as long as the NF-κB/NF-AT binding motif remains unaltered. This motif may also include the zinc finger domain. A logical region that may be targeted by the amino-terminal proline-rich region of Foxp3 is the Rel homology domain found in both NF-κB and NF-AT family proteins. A region that may also be important with respect to Foxp3 function is the leucine zipper domain, as demonstrated by the number of mutations associated with IPEX that have been mapped in this region of Foxp3. The role of this domain in Foxp3 function remains uncharacterized, but may play a role in dimer formation as it does in other Foxp family members [44].

Because the pathogenesis of a number of retroviral-induced immunologic disorders such as HIV-1/AIDS and HTLV-I/HAM/TSP have been associated with dysregulation of Foxp3 expression [8,45], we also examined the role of Foxp3 in retroviral gene expression. HIV-1 LTR activation in CD4^+^ T cells is critically dependent on two tandem NF-κB sites located between nucleotide positions −102 and −81 within the HIV-1 enhancer region, whereas HTLV-I LTR activation in the presence or absence of the HTLV-I-encoded transactivator protein Tax is independent of NF-κB [18]. To our knowledge for the first time, Foxp3 was shown to have a direct effect on HIV-1 LTR transcription. Deletion of the NF-κB sites within the HIV-1 enhancer region reduced the responsiveness of the HIV-1 LTR to Foxp3-mediated suppression. In addition, the FKH domain of Foxp3 was required for this inhibitory effect in HEK 293T cells, but not in Jurkat T cells, similar to Foxp3-mediated suppression of a synthetic NF-κB reporter. The direct effect of Foxp3 down-regulating HIV-1 gene expression correlates well with recently reported evidence indicating that higher regulatory activity of CD4^+^CD25^+^ T cells from HIV-1-infected patients was associated with lower HIV-1 viral loads in these patients [46].

Foxp3 also affected two well-known functions of HTLV-I Tax: transactivation of the NF-κB pathway and, most surprisingly, transactivation of the HTLV-I LTR. Transactivation of the HTLV-I LTR by Tax involves the interaction of ATF/CREB factors with Tax in the nucleus. Binding of Tax enhances ATF/CREB dimerization and promotes assembly of Tax-ATF/CREB complexes onto specific sequences in the viral promoter known as Tax-responsive elements. This series of steps allows Tax to recruit coactivator proteins CBP/p300 to the viral promoter and facilitate a high level of viral gene expression [24–26]. Transactivation of the NF-κB pathway by Tax was inhibited by overexpression of full-length Foxp3, but not ΔFKH, as seen with basal activation of the HIV-1 LTR and a synthetic NF-κB reporter in HEK 293T cells. However, Tax-mediated transactivation of the HTLV-I LTR was inhibited by overexpression of both full-length Foxp3 as well as ΔFKH in both HEK 293T cells and CD4^+^ T cells. We demonstrated that Foxp3 did not directly affect the functioning of Tax, but rather Foxp3 targeted the transcription factors required for Tax transactivation (i.e., NF-κB and a then-unknown cellular factor, which we identified in this study as CREB). The negative effect of Foxp3 on HTLV-I gene expression was confirmed utilizing an HTLV-I infectious molecular clone.

Importantly, we demonstrated that HTLV-I-infected individuals with the highest levels of Foxp3 protein expression within the CD4^+^CD25^+^ T cells population exhibited lower proviral loads than did individuals with the lowest levels of Foxp3 protein expression. Previous studies have demonstrated that the HTLV-I proviral load directly correlates with HTLV-I Tax mRNA load, the frequency of immunopathogenic virus-specific CD8^+^ T cells, and disease severity in patients with HAM/TSP [47]. These results have important implications on the utility of Foxp3 in controlling viral gene expression and thus pathogenesis of HAM/TSP. Therefore, Foxp3 becomes an attractive target for the development of novel therapeutic applications directed at modulating the expression of this important regulatory protein, especially in light of recent observations that the expression of Foxp3 can also be down-regulated by HTLV-I Tax [8].

As the activation of the HTLV-I LTR depends primarily on ATF/CREB proteins (whether in the presence or the absence of Tax), we investigated whether Foxp3 could interact with this additional cellular signaling pathway. While the DNA-binding activity of CREB is, in most cases, constitutive, the transactivation potential of CREB is regulated by the phosphorylation of CREB and recruitment of CBP/p300 [48]. Our data demonstrate that Foxp3 interferes with the latter of these two processes and that the recruitment of the coactivator protein p300, and resulting transcriptional activation are blocked by Foxp3. This may be the result of the physical interaction we detected between Foxp3 and p300. With respect to HTLV-I LTR activity, while full-length Foxp3 inhibited both basal and Tax-dependent transcription by ~50%, ΔFKH appeared less effective in suppressing basal activation (~25% inhibition) compared to Tax-dependent activation (~50% inhibition). The effect of ΔFKH on basal activation of the HTLV-I LTR in HEK 293T cells was very similar to that shown for a synthetic CREB reporter, suggesting that the FKH domain of Foxp3 is important at some level. As observed with NF-κB activation, the Foxp3 mutant lacking the FKH domain was a stronger inhibitor of CREB activation in CD4^+^ T cells than in HEK 293T epithelial cells. Therefore, it appears that in CD4^+^ T cells, the FKH domain is dispensable for the proper functioning of Foxp3 with respect to both NF-κB and CREB activation.

In summary, this is, to our knowledge, the first direct evidence implicating a role for the Treg-specific transcription factor Foxp3 in regulating retroviral gene expression. In addition, we identify the CREB pathway as a molecular target of Foxp3. Since CREB has been shown to regulate multiple genes involved in transcription (e.g., JunD, c-Fos, signal transducer of activated T cells 3 [STAT3]), cell cycle (e.g., p15^INK4b^, cyclin A, cyclin D1), and immune regulation (e.g., IL-2, IL-6, T-cell receptor α) (reviewed in [48]), the findings presented in this report broaden the potential range of signaling pathways under the control of the regulatory protein Foxp3. Our evidence stresses the importance of Foxp3 expression and Treg function in the development and maintenance of protective immunity against HIV-1 and HTLV-I. Based on recent findings, Foxp3 may limit HIV-1 and HTLV-I transcription by interfering with activation of NF-κB and CREB pathways. However, observing that this inhibitory effect is not absolute, a low level of viral gene expression may persist in CD4^+^ T cells (in particular regulatory T cells, which are known reservoirs of HIV-1 and HTLV-I) and result in the accumulation of viral proteins that either stimulate NF-κB and/or CREB activation or directly inhibit Foxp3 expression or function. The imbalance of NF-κB and CREB activation caused by these viral gene products may be a crucial step in the pathogenesis of virus-induced immunological disorders such as AIDS and HAM/TSP. Future studies will be directed at identifying and characterizing cellular proteins that interact with Foxp3 both in the nucleus and cytoplasm, in order to better address how Foxp3 functions to guide the development and function of regulatory T cells in health and disease.

## Materials and Methods

### Cell culture.

HEK 293T cells were cultured in Dulbecco's modified Eagle medium (Invitrogen, Carlsbad, California, United States). Jurkat T cells and primary human CD4^+^ T cells were cultured in RPMI-1640 medium (Invitrogen). Media were supplemented with 2 mM L-glutamine, 100 U/ml penicillin, 100 μg/ml streptomycin (Cambrex, East Rutherford, New Jersey, United States), and 10% fetal bovine serum (Atlanta Biologicals, Norcross, Georgia, United States).

### Patients and cell preparation.

PBMCs were prepared by centrifugation over Ficoll-Hypaque gradients (BioWhittaker, Walkersville, Maryland, United States) from eight HAM/TSP patients and eight ACs, and the cells were viably cryopreserved in liquid nitrogen until tested. HAM/TSP was diagnosed according to WHO guidelines [49]. HTLV-I seropositivity was determined by ELISA (Abbott Laboratories, Abbott Park, Illinois, United States), with confirmation by Western blot analysis (Genelabs Technologies, Redwood City, California, United States). Blood samples were obtained after informed consent as part of a clinical protocol reviewed and approved by the NIH institutional review panel.

### Plasmids.

Expression vectors encoding human Foxp3 (pCMV-Foxp3-IRES-EGFP) and human Foxp3 lacking the forkhead (FKH) domain (pCMV-ΔFKH-IRES-EGFP) were generous gifts from S. Ziegler (Benaroya Research Institute). pEGFP-C2 was provided by I. Lipinski (NIDDK/NIH). pcDNA3 was provided by K. T. Jeang (NIAID/NIH). pCMV4-Tax was a generous gift from W. Greene (University of California San Francisco). pGL4-luc2 and pGL4-TKhRluc2 were purchased from Promega (Madison, Wisconsin, United States). pUC18 was purchased from Stratagene (La Jolla, California, United States). HIV-1 wt LTR and HIV-1 Δ-κB LTR luciferase reporter vectors were constructed by cloning the XhoI/HindIII LTR fragments from pHIV-CAT and pΔ-κB-HIV-CAT (AIDS Research and Reference Reagent Program, NIAID/NIH) into the multiple cloning site of pGL4-luc2. NF-κB, HTLV-I LTR, and CREB luciferase reporter and pCMV-p300-HA expression vectors were generously provided by B. Wigdahl (Drexel University College of Medicine). HTLV-I pACH infectious molecular clone has been described previously [30]. pFR-luc, pFA-CMV, pFA2-CREB-1, and pFA2-c-Jun were purchased from Stratagene. pFA-Tax (encoding a fusion protein consisting of the Gal4 DNA-binding domain fused in-frame to HTLV-I Tax) was constructed by PCR amplification of HTLV-I Tax using pCMV4-Tax as a template and BamHI/BglII-tagged primers. The amplified insert was digested and ligated into the BamHI/BglII sites of pFA-CMV. Plasmid contents were confirmed by DNA sequencing.

### Isolation of primary human CD4^+^ T cells.

CD4^+^ T cells were isolated from cryopreserved healthy donor PBMCs by negative selection with the CD4^+^ T Cell Isolation Kit II (Miltenyi Biotech, Bergisch Gladbach, Germany) according to manufacturer's guidelines. Purity of negatively selected CD4^+^ T cells was consistently higher than 96% as determined by flow cytometry.

### Transient expression and luciferase assays.

HEK 293T cells were plated at a density of 5 × 10^5^ cells/well in six-well culture plates (BD Biosciences, San Diego, California, United States) 1 d prior to transfection with the appropriate plasmid DNA (~2 μg total) using FuGene 6 transfection reagent (Roche, Basel, Switzerland). Jurkat T cells were plated at 1 × 10^6^ cells/well in six-well culture plates the day of transfection with the appropriate plasmid DNA (~2 μg total) using FuGene 6 transfection reagent. Primary human CD4^+^ T cells (2 × 10^6^) were nucleofected with the specified plasmid DNA (5 μg total) using the Human T Cell Nucleofection Kit (Amaxa, Gaithersburg, Maryland, United States). Forskolin (10 μM; Calbiochem, San Diego, California, United States) was added in some experiments 20 h posttransfection. Cells were harvested 24 h posttransfection and luciferase activity was analyzed using the Dual-luciferase Reporter Assay System (Promega) and a Monolight 2010 luminometer (Analytical Luminescence Laboratory, San Diego, California, United States) according to manufacturer's guidelines. pGL4-TKhRluc2 was used as an internal control to normalize for transfection efficiency. Nucleofected CD4^+^ T cells were also monitored for transfection efficiency and cell viability 24 h posttransfection as follows. Transfection efficiency was routinely ~30% as determined by flow cytometric analysis of EGFP expression. Cell viability, determined by staining with 7-amino-actinomycin D (7-AAD; BD Biosciences), was routinely ~70%. Both transfection efficiency and cell viability in nucleofected CD4^+^ T cells was independent of the plasmids used.

### Foxp3 and HTLV-I Tax expression analysis by real-time RT-PCR.

Real-time RT-PCR analysis of Foxp3 and HTLV-I Tax expression was performed as previously described [8,47]. Briefly, total RNA was extracted using the RNeasy Mini Kit (Qiagen, Valencia, California, United States) according to manufacturer's guidelines, and cDNA was synthesized by reverse transcription using TaqMan Gold RT-PCR Kit using random hexamer primers (Applied Biosystems, Foster City, California, United States). Foxp3 and HTLV-I Tax mRNA expression was quantified by real-time PCR using ABI PRISM 7700 Sequence Detection System (Applied Biosystems). The normalized values in each sample were calculated as the relative quantity of Foxp3 or HTLV-I Tax mRNA expression divided by the relative quantity of HPRT mRNA expression. The values were calculated by the following formula: normalized Foxp3 or HTLV-I Tax expression = 2^Ct value of HPRT – Ct value of Foxp3 or HTLV-I Tax^.

### Real-time PCR.

Real-time PCR analysis of HTLV-I (Tax) proviral load was performed as previously described [47,50]. DNA was extracted from 1 × 10^6^ cells using Puregene DNA Isolation Kit (Gentra, Minneapolis, Minnesota, United States), and 100 ng of the sample DNA solution was analyzed by this system. The HTLV-I proviral DNA load was calculated by the following formula: copy number of HTLV-I (*pX*) per 100 cells = (copy number of *pX*)/(copy number of β-actin/2) × 100.

### Western blot analysis.

HEK 293T cells were plated at a density of 5 × 10^5^ cells/well in six-well culture plates (BD Biosciences) 1 d prior to transfection with the appropriate plasmid DNA (2 μg total) using FuGene 6 transfection reagent (Roche). Cells were harvested 24 h posttransfection for whole-cell lysates in RIPA buffer (50 mM Tris-HCl [pH 7.4], 150 mM NaCl, 1% Igepal (NP-40), 0.5% sodium deoxycholate, 1 mM EDTA, 1 mM DTT, 1 mM PMSF, and 1× Complete Mini Protease Inhibitor [Roche]). Protein concentration was determined by Lowry assay (Bio-Rad, Hercules, California, United States) and colorimetric reactions were read using a VersaMax microplate reader (Molecular Devices, Sunnyvale, California, United States) at an absorbance of 750 nm. Size fractionation was performed on 20 μg of protein/sample by SDS-PAGE, and the protein was transferred to nitrocellulose or PVDF membranes and subjected to immunoblotting using the indicated antibodies. Foxp3 was detected using rabbit anti-human Foxp3 polyclonal antibody (ab4728 or ab10563; Abcam, Cambridge, United Kingdom) and anti-rabbit IgG-HRP secondary antibody (Cell Signaling Technology, Beverly, Massachusetts, United States). NF-κB p65 and CREB-1 were detected using rabbit anti-human polyclonal (p65) or monoclonal antibody (CREB-1; 48H2) (Cell Signaling Technology). Beta-actin was detected using a mouse monoclonal antibody (AC-15; Sigma, St. Louis, Missouri, United States). For coimmunoprecipitation analysis, cell lysates were precleared with 30 μl of protein A/G plus-agarose beads (Santa Cruz Biotechnology, Santa Cruz, California, United States) and then incubated with mouse monoclonal anti-HA antibody (6E2; 1:100; Cell Signaling Technology) and 30 μl of protein A/G plus-agarose beads overnight. The immunoprecipitates were washed four times with RIPA buffer, resuspended in SDS sample buffer, and heated at 95 °C for 5 min. Proteins were then treated as described for Western blot analysis.

### Transcription factor DNA-binding analysis (TF-ELISA).

CREB-1 and ATF-2 DNA-binding activity was analyzed with the TransFactor Profiling (Inflammation 1) Kit (BD Biosciences) according to the manufacturer's protocol. Nuclear extracts were prepared from HEK 293T cells transfected with a control vector (EGFP) or Foxp3 expression vector (1,000 ng) in the presence or absence of forskolin (10 μM for 4 h) using the TransFactor Extraction Kit (BD Biosciences). Protein concentration was determined using a Biophotometer (Eppendorf, Hamburg, Germany). Nuclear extracts (20 μg) were incubated in preblocked wells containing plate-bound double-stranded oligonucleotides corresponding to an ATF/CREB consensus sequence (…TGACATCA…). Wells were washed, incubated with the appropriate primary antibody, washed, incubated with secondary antibody (HRP-labeled), washed again, and finally developed with TMB substrate. Colorimetric reactions were read using a VersaMax microplate reader (Molecular Devices) at an absorbance of 655 nm.

### Flow cytometric analysis of Foxp3 protein expression.

Cryopreserved PBMCs from HAM/TSP patients or HTLV-I-infected ACs were thawed and washed with FACS buffer (1× PBS, 0.1% NaN_3_, 5% FBS). Cells (1.5 × 10^6^) were fixed by sequential formaldehyde/methanol fixation as follows. Cells were carefully resuspended in FACS buffer and fixed with 100 μl of reagent A (Fix & Perm kit; Caltag Laboratories, Burlingame, California, United States) at room temperature for 3 min followed by 2 ml of 70% methanol for 5 min at 4 °C. Cells were washed twice and permeabilized with 100 μl of reagent B (Fix & Perm kit) and stained for intracellular Foxp3 with mouse anti-human Foxp3 monoclonal antibody (0.5 μg of ab22510; Abcam) or the appropriate isotype control for 30 min. Cells were washed twice and stained with Cy5-conjugated goat anti-mouse immunoglobulin F(ab′)2 secondary antibody (Caltag Laboratories) for an additional 30 min. Cells were washed twice and stained for surface CD4 expression with PE-labeled anti-CD4 (BD) and CD25 expression with FITC-labeled anti-CD25 (BD). Cells were washed twice and analyzed on a FACSCalibur (BD). Data analysis was performed using FlowJo (Tree Star, Ashland, Oregon, United States).

### Statistical analyses.

The Mann-Whitney U test was used to compare the data between patients with HAM/TSP and AC.

## Abbreviations

AC: asymptomatic carrier
ATF: activating transcription factor
CBP: CREB-binding protein
CREB: cAMP-responsive element binding protein
EGFP: enhanced green fluorescent protein
FKH: forkhead
HAM/TSP: HTLV-I-associated myelopathy/tropical spastic paraparesis
HTLV-1: human T cell lymphotropic virus type I
IPEX: immune dysregulation, polyendocrinopathy, enteropathy, X-linked syndrome
LTR: long terminal repeat
NF-AT: nuclear factor of activated T cells
NF-κB: nuclear factor-κB
Treg: T regulatory cell
